# The specificity of emotion dysregulation in adolescents with borderline personality disorder: comparison with psychiatric and healthy controls

**DOI:** 10.1186/s40479-017-0052-x

**Published:** 2017-01-10

**Authors:** Marina Ibraheim, Allison Kalpakci, Carla Sharp

**Affiliations:** Department of Psychology, University of Houston, 126 Heyne Building, Houston, TX 77204 USA

**Keywords:** Borderline personality disorder, Adolescents, Emotion dysregulation

## Abstract

**Background:**

Research has supported the notion that emotion dysregulation is a core feature of BPD. However, given that this feature is typical of healthy adolescents as well as adolescents with other psychiatric disorders, the specificity of emotion dysregulation to BPD in this age group has not yet been determined. The overall aim of this study was to examine emotion dysregulation in adolescent inpatients with BPD compared with non-BPD inpatient adolescents and healthy non-clinical adolescents, taking into account both global emotion dysregulation deficits and more specific impairments.

**Method:**

The sample included 185 adolescent inpatients with BPD (*M* = 15.23, SD = 1.52), 367 non-BPD psychiatric inpatient adolescents (*M* = 15.37, *SD* = 1.40), and 146 healthy adolescents (*M* = 15.23, *SD* = 1.22), all of whom were between the ages of 12 and 17. Borderline personality features were assessed, along with emotion dysregulation and psychiatric severity.

**Results:**

After controlling for age, gender, and psychiatric severity, results revealed that adolescents with BPD had higher overall emotional dysregulation compared with non-BPD psychiatric controls and healthy controls. These differences were apparent in only two domains of emotion dysregulation including limited access to emotion regulation strategies perceived as effective and impulse control difficulties when experiencing negative emotions.

**Conclusions:**

Findings suggest BPD-specific elevations on emotion dysregulation generally, and subscales related to behavioral regulation specifically.

## Background

Adolescence is a time of social, physical, cognitive and emotional change [1, 2]. Socially, adolescents establish more relationship types than in childhood [3], effectively widening their social circle. In adolescence, the frontal lobe, responsible for judgment and inhibition is underdeveloped [4] relative to the limbic system, which is responsible for emotional processing [5]. Thus, when confronted with arousing emotional situations, there is an “activation of strong drives, appetites, emotional intensity, and sensation seeking,” that cannot be regulated easily [5]. For these reasons, adolescents exhibit greater difficulties in emotion dysregulation when compared to adults and children [5–7].

In this context, emotion dysregulation can be generally defined as “the frequent and intense experience of emotions combined with an inability to cope with their occurrence” [8]. Gratz and Roemer [9] operationalized emotion dysregulation as a multidimensional construct, encompassing emotional awareness, understanding, and acceptance of one’s emotions, in addition to the ability to manage emotional arousal and to act “in desired ways regardless of emotional state.” Within this broad conceptualization, Gratz and Roemer proposed six sub-factors of emotion dysregulation: lack of awareness of emotional responses, lack of clarity of emotional responses, non-acceptance of emotional responses, limited access to emotion regulation strategies perceived as effective, difficulties controlling impulses when experiencing negative emotions, and difficulties engaging in goal-directed behaviors when experiencing negative emotions [9]. Though interrelated, these sub-factors are thought to be conceptually distinct. Indeed, this multidimensional structure has been validated across multiple samples, including adolescents, and in clinical [10] and non-clinical groups [11, 12].

While some degree of emotion dysregulation is typical for adolescents, emotion dysregulation may be indicative of psychiatric problems. Indeed, research has shown that adolescents with high levels of emotion dysregulation have been found to have elevated rates of depression [13, 14], anxiety [15, 16], substance use [17], conduct problems [18], attention-deficit/hyperactivity disorder [19] and suicidal and self-harming behaviors [20]. A prototypical example in this regard is adolescents who suffer from borderline personality disorder (BPD). BPD is a serious psychiatric disorder characterized by impulsivity, mood instability, and relationship instability [21]. Some have questioned whether the disorder can be diagnosed in adolescence, but recent research has provided evidence for the validity of the diagnosis in this age group [22–26]. Theories on the development of BPD emphasize that problems with emotion dysregulation are a core, underlying feature of BPD [27, 28]. Indeed, this conceptualization has become so widely accepted that affective instability has become one of the nine defining criteria of BPD [26]. Empirical research echoes theoretical perspectives demonstrating a BPD diagnosis in adolescents [29–34] and BPD symptoms [27, 35] associate with problems in emotion dysregulation [36]. It is worth noting that these findings have been supported by data obtained from self-reports [29, 31, 35] and from behavioral studies [30, 32, 33], with one study taking care to utilize both behavioral and self-report data in order to support this claim [34].

Taken together, research and clinical nomenclature supports the notion that emotion dysregulation is characteristic of BPD [12]; however, there are some aspects that current research has not yet addressed. First, no research has simultaneously compared adolescents with BPD to psychiatric control adolescents and healthy controls. Since no study has compared these three groups to each other simultaneously, it is unclear if and how emotion dysregulation in BPD differs from emotion dysregulation in adolescents with non-BPD psychiatric disorders and typical adolescents. Second, no study—to our knowledge—has compared these three groups on the six sub-factors of emotion dysregulation as proposed by Gratz and Roemer [9]. We know from previous studies that adolescents with BPD exhibit greater emotion dysregulation than their typical adolescent counterparts [37], but it is unclear whether certain emotion dysregulation sub-factors are more characteristic of this group than others. Therefore, more research is needed to pinpoint on which specific sub-factors of emotion dysregulation these three adolescent groups differ. Finally, most studies have not considered the potential confounding effects of gender [38], age [39, 40], and general psychiatric severity [41], as each of these has been found to correlate with BPD and emotion dysregulation, and thus may obscure the true relation between BPD and emotion dysregulation in this age group.

Against this background, the overall aim of this study was to examine emotion dysregulation in adolescent inpatients with BPD compared with non-BPD inpatient adolescents and healthy non-clinical adolescents recruited from the community. We compared adolescent inpatients with BPD to non-BPD psychiatric controls and non-clinical adolescent healthy controls on total score of emotion dysregulation and the six aforementioned emotion dysregulation sub-factors proposed by Gratz and Roemer [9]. Given that previous research has identified significant relations between emotion dysregulation and gender [38] and age [39], we controlled for these variables in the study analyses. Moreover, previous research has demonstrated a relation between degree of psychiatric severity and level of emotion dysregulation [41], and thus we controlled for psychiatric severity to ensure that differences among the groups in emotion dysregulation were due to BPD pathology specifically, rather than psychiatric severity, generally. Based on findings from previous research [42], and developmental theories of BPD [27, 28], we hypothesized that adolescents with BPD would exhibit higher levels of overall emotion dysregulation [11, 27, 43], as well as on specific sub-factors of emotion dysregulation, than both psychiatric and healthy controls, controlling for gender, age, and psychiatric severity.

## Methods

### Participants

The clinical sample was recruited through an inpatient psychiatric hospital in an ongoing research study. Exclusion criteria included severe aggression, active psychosis, IQ <70, and/or non-English speaking. At the time of admission, licensed clinicians were consulted to assess whether patients were stable enough to participate. If there was evidence of cognitive deficits or psychosis, neuropsychological testing was completed by a licensed staff psychologist to determine whether that adolescent should be excluded from the study. Of 711 consecutive admissions to the hospital, 39 were excluded based on aforementioned criteria. Of the remaining patients who were approached for consent, 52 declined participation, three revoked consent, and 30 were excluded based on information obtained after consent was given. Additionally, 35 participants were excluded to missing data on main study variables. A total of 552 inpatient adolescents were given an interview-based measure of BPD. *N* = 187 (33.8.%) adolescent inpatients met criteria for DSM-IV BPD and *n* = 365 adolescent inpatients did not meet criteria for BPD and constituted the non-BPD psychiatric control group.

Healthy adolescents were recruited through schools and community resources. Adolescents were excluded if they met diagnostic criteria for any psychiatric disorder. A total of *N* = 223 adolescents consented for participation in the present study, of which *n* = 34 failed to attend their scheduled appointments and *n* = 3 were excluded based on the aforementioned exclusion criterion. Additionally, 40 participants were excluded due to missing data. Therefore, the final sample consisted of *N* = 146 participants. Participant characteristics and psychiatric comorbidity are presented in Table 1.

**Table 1:** Sample Characteristics

	BPD (*n* =185, 26.5%)		Non-BPD psychiatric (*n* = 367, 52.6%)		Healthy (*n* = 146, 20.9%)	
	*n* or *M*	*%* or (*SD*)	*n* or *M*	*%* or (*SD*)	*n* or *M*	*%* or (*SD*)
Age	15.23	(1.52)	15.37	(1.40)	15.23	(1.22)
Female	149	80.50%	200	54.50%	105	71.90%
Hispanic	17	10.30%	16	4.90%	41	28.10%
Race						
Caucasian	138	84.70%	290	89.50%	15	10.90%
African American	3	1.80%	6	1.90%	33	24.10%
Asian	5	3.10%	13	4%	53	38.70%
American Indian/Alaskan Native	1	0.60%	0	0.00%	6	4.4%
Multiracial or other	16	9.80%	15	4.60%	30	21.9%
Disorder						
Depressive	120	72.70%	160	47.60%	-	-
Bipolar	22	13.30%	13	3.90%	-	-
Eating	23	13.90%	18	5.30%	-	-
Externalizing	97	58.40%	117	34.70%	-	-
Anxiety	122	73.10%	171	50.40%	-	-

*Note.* BPD Diagnoses were based on the Childhood Interview for Borderline Personality Disorder (CI-BPD, [44]). Other psychiatric disorders diagnoses were based on the Computerized Diagnostic Interview Schedule for Children [73]. Prevalence rates are exclusively with regard to positive diagnoses in which the adolescent endorsed all necessary diagnostic criteria. Depressive Disorder includes Major Depressive Disorder and Dysthymia; Bipolar Disorder includes mania and hypomania; Eating Disorder includes Bulimia Nervosa and Anorexia Nervosa; Anxiety Disorder includes Generalized Anxiety Disorder, Separation Anxiety Disorder, Social Phobia, Specific Phobia, Obsessive Compulsive Disorder, Panic Disorder, Agoraphobia, and Post Traumatic Stress Disorder; Externalizing Disorder includes Conduct Disorder, Oppositional Defiant Disorder, and Attention Deficit Hyperactivity Disorder

### Measures

#### Emotion dysregulation

*Difficulties in Emotion Regulation scale* (DERS, [9]). The DERS is a self-report measure containing 36 items that assess the following six aspects of emotion regulation: nonacceptance of emotion responses, difficulties in engaging in goal-directed behavior, impulse control difficulties, lack of emotion awareness, limited access to emotion regulation strategies, and lack of emotional clarity. Each of these aspects has its own separate scale score. Each item on the DERS was rated on a 5-point Likert scale from 1 (*almost never [0–10%]*) to 5 (*almost always [91–100%]*), so higher scores indicate greater difficulties in emotion regulation. This measure has previously demonstrated adequate psychometric properties in both clinical [10] and community [11, 12] adolescent samples. In the present study, Chronbach’s alpha for the total score was α = .96. In addition, each subscale had good internal consistency (α: nonacceptance = 0.92, goals = 0.87, impulse = 0.84, awareness = 0.85, strategies = 0.93, clarity = 0.87).

#### Borderline personality disorder

*Childhood Interview for DSM-IV Borderline Personality Disorder* (*CIBPD*, [44])*.* The CIBPD was adapted from the borderline module of the Diagnostic Interview for DSM-IV Personality Disorders (DIPD-IV, [45]). It is a semi-structured interview used specifically to assess BPD in adolescents by assessing the following DSM-IV criteria for BPD: symptoms of inappropriate anger, affective instability, chronic feelings of emptiness, identity disturbance, transient stress-related paranoid ideation or severe dissociative symptoms, fears of abandonment, recurrent suicidality or self-harm behavior, impulsivity, and intense interpersonal relationships. Trained interviewers rated symptoms using “0” for absence of symptom, “1” if the symptom is probably present, or “2” if the symptom is definitely present. A full diagnosis of BPD requires a score of 2 for on least five of the nine criteria. A dichotomous score on the CIBPD was used in the analyses to determine a diagnosis of BPD. In addition, a dimensional CIBPD score, which reflects the total count of ratings of the nine symptoms (i.e. 0–18) was used. In the current study, inter-rater reliability was conducted with 12% of the sample, with two raters, with Kappa’s ranging from good (ĸ = 0.77; *p* < .001) to very good (ĸ = 0.89; *p* < .001) agreement. Inter-rater reliability values were obtained for the psychiatric control and BPD groups only.

#### Psychiatric severity

*Youth Self-Report* (YSR, [46]) measures psychopathology. It is a self-report that contains 112 items, each scored on a three-point scale using 0 as “not true”, 1 as “somewhat or sometimes true”, and 2 as “very or often true”. The measure has a Total Problems T-score of general psychiatric functioning and two subscales: Externalizing Behavior Problems and Internalizing Behavior Problems. Our study will look at the Total Problems T-score.

### Procedures

Approval for this study was obtained from local institutional review boards. For all participants, trained research coordinators and clinical psychology graduate students administered self-report assessments and conducted interviews under the direct supervision of the senior author. Clinical adolescents completed assessments during the first 2 weeks of their hospitalization. Non-clinical adolescents completed assessments during a scheduled assessment day. The Principal Investigator of the study met monthly with the research team to review interview-based assessments for reliability and training.

### Data analytic strategy

The data analyses involved several steps. First, descriptive analyses on main study variables, including calculations of means and standard deviations, were performed. Next, to examine the bivariate relations among main study variables, an analyses of variance (ANOVA) was conducted to compare the three groups on psychiatric severity (as measured by scores on YSR total problems) and age. Given that three groups were being compared, a post-hoc Fisher’s LSD test was performed in order to restrict the family-wise error rate to alpha. Next, a Pearson chi-square test was conducted to determine whether adolescents with BPD were more likely to be male or female.

Then, to determine whether there were differences across groups in emotion dysregulation, controlling for covariates, an analysis of covariance (ANCOVA) was performed with group (BPD, psychiatric control, healthy control) as independent variable and DERS total score and covariates (gender, age, and psychiatric severity) as dependent variables. If findings revealed that groups differed on emotion dysregulation, a Fisher’s LSD post-hoc test was conducted to clarify differences among the groups with regard to the DERS total score. Additionally, given the focus on dimensional approaches to personality pathology, we reran analyses using continuous CIBPD and DERS total to confirm the categorical findings.

Finally, a multivariate analysis of covariance (MANCOVA) was conducted to compare the three groups on the DERS subscale scores, controlling for the effects of gender, age, and psychiatric severity. If results revealed that group predicted DERS total score at the multivariate level, we further examined findings at the univariate level and conducted post-hoc Fisher’s LSD tests to elucidate differences among the groups with regard to specific DERS subscales.

## Results

Descriptive analyses for DERS total and subscale scores as well as YSR total problems across adolescents with BPD, non-BPD psychiatric controls, and healthy controls are provided in Table 2. An analysis of variance (ANOVA) was conducted to compare the three groups on psychiatric severity as measured by scores on YSR total problems. Results revealed a significant difference across groups, *F*(2, 677) = 202.26, *p* < .001. A Fisher’s LSD post-hoc test revealed that adolescents with BPD (*M* = 71.03, *SD* = 8.45) had higher scores on YSR Total Problems than non-BPD psychiatric controls (*M* = 62.24, *SD* = 9.74, *p* < .001 and healthy controls (M = 49.22, SD = 11.00, *p* < .001). Psychiatric controls also had higher scores on YSR Total Problems than healthy controls, *p* < .001.

**Table 2:** DERS and YSR scores across inpatients adolescents with BPD, non-BPD psychiatric controls, and healthy controls

	BPD (*n* =187, 26.5%)		Non-BPD psychiatric (*n* = 365, 52.6%)		Healthy (*n* = 146, 20.9%)	
	*M*	(*SD*)	M	(*SD*)	M	(*SD*)
DERS Total score	121.69	(23.43)	99.20	(28.65)	72.66	(21.81)
Nonacceptance of emotional responses	18.06	(6.71)	14.08	(6.88)	10.79	(4.80)
Difficulty engaging in goal directed	20.20	(4.51)	17.38	(5.28)	13.23	(5.20)
Impulse control	19.35	(6.25)	14.30	(6.57)	9.63	(4.68)
Lack of emotional awareness	18.94	(5.93)	17.73	(5.92)	14.56	(5.16)
Limited access to emotion regulation strategies	29.08	(7.07)	22.07	(8.84)	14.65	(6.73)
Lack of emotional clarity	16.05	(4.98)	13.64	(5.17)	9.80	(4.02)
YSR total score	71.03	(8.45)	62.24	(9.74)	49.22	(11.00)

*Note. DERS* difficulty in emotion regulation scale, *YSR* youth self-report. Values are means that have not been adjusted to control for the potentially confounding effects of gender, age, and psychiatric severity

A Pearson chi-square test revealed that adolescents with BPD were more likely to be female than non-BPD psychiatric controls and healthy controls, *Χ* ^*2*^ = 40.53, *p* < .001. Results from an ANOVA showed that groups did not differ significantly on age *F*(2, 695) = .88, *p* = .414.

### Comparison of DERS total scores across BPD adolescents, non-BPD psychiatric controls, and healthy controls

**Fig. 1:**
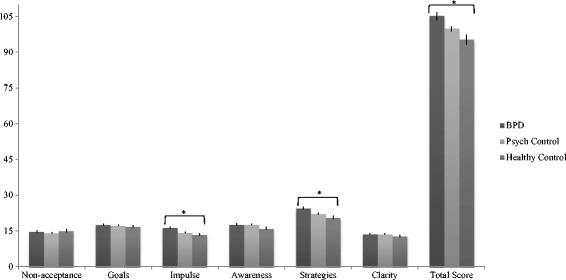
DERS total and subscale scores across adolescents with BPD, non-BPD psychiatric controls, and healthy controls

An analysis of covariance (ANCOVA) was conducted to compare the three groups on the DERS total score, controlling for the effects of gender, age, and psychiatric severity. As shown in Fig. 1, results demonstrated a significant effect of group, *F*(1, 647) = 4.40, *p* = .013, *η*_*p*_^*2*^ = .013.

As shown in Table 2, a Fisher’s LSD post-hoc test revealed that, controlling for covariates, adolescent inpatients in the BPD group (*M* = 121.69, *SD* = 23.43) had significantly higher DERS total scores than adolescents in the Healthy controls group (*M* = 72.66, *SD* = 21.81), *p* = .016. Adolescents in the BPD group also had significantly higher total mean DERS score than the non-BPD psychiatric control group (*M* = 99.20, *SD* = 28.65), *p* = .031. Adolescents in the non-BPD psychiatric group and healthy control group did not differ on their mean DERS total score, *p* = .549.

### The relation between CIBPD dimensional score and DERS total score

To confirm the categorical analysis above, multiple regression analysis was conducted to examine whether CIBPD dimensional score predicted DERS total score, controlling for gender, age, and psychiatric severity. Results indicated that the predictors explained 59% of the variance (*R* ^2^ = .59, F[4, 646] = 235.36, *p* < .001). Specifically, CIBPD dimensional score significantly predicted the DERS total score (β = .19, *p* < .001), as did psychiatric severity (β = .62, *p* < .001) and gender (β = −.09, *p* < .001), such that females (*M* = 102.40, *SD* = 31.56) had higher self-reported DERS total scores than males (*M* = 96.66, *SD* = 27.53) scores, *t*(696) = 2.53, *p* = .01. Age did not significantly predict the DERS total score.

### Comparison of DERS subscales across BPD adolescents, non-BPD psychiatric controls, and healthy controls

A multivariate analysis of covariance (MANCOVA) was conducted to compare the three groups on the DERS subscale scores, controlling for the effects of gender, age, and psychiatric severity. As shown in Fig. 1, results demonstrated, at the multivariate level, a significant effect of group on the dependent variables, Wilks’ λ = .95, *F*(12, 1284) = 2.79, *p* = .001, *η*_*p*_^*2*^ = .03.

At the univariate level, the groups differed on the DERS subscale of impulse control difficulties, *F*(2, 167.77) = 5.72, *p* = .003, *η*_*p*_^2^ = .02. A Fisher’s LSD test revealed that adolescents with BPD (*M* = 19.35, *SD* = 6.25) had higher mean scores on this subscale compared with non-BPD psychiatric controls (*M* = 14.30, *SD* = 6.57; *p* = .001) and healthy controls (*M* = 9.63, *SD* = 4.68; *p* = .006). Groups also differed on the DERS subscale of limited access to emotion regulation strategies, *F*(2, 315.29) = 7.12, *p* = .001, *η*_*p*_^*2*^ = .02. A Fisher’s LSD post-hoc test revealed that adolescents with BPD (*M* = 29.08, *SD* = 7.07) had significantly higher mean scores on this scale compared with non-BPD psychiatric controls (*M* = 22.07, *SD* = 8.84; *p* = .001) and healthy controls (*M* = 14.65, *SD* = 6.73; *p* = . 001). No other DERS subscale differed across the three groups.

## Discussion

The overall aim of this study was to examine emotion dysregulation in adolescent inpatients with BPD compared with non-BPD inpatient adolescents and healthy non-clinical adolescents. More specifically, we compared adolescent inpatients with BPD to non-BPD psychiatric controls and non-clinical adolescent healthy controls on total levels of emotion dysregulation and the six aforementioned emotion dysregulation sub-factors proposed by Gratz and Roemer [9]. After controlling for age, gender, and psychiatric severity, we found that adolescents with BPD had higher overall emotional dysregulation compared with both non-BPD psychiatric controls and healthy controls. Analyses that examined the relation between dimensional scores of BPD and emotion dysregulation confirmed this finding. With regard to specific DERS subscales, adolescents with BPD had higher self-reported scores than both groups on Limited Access to Strategies and Impulse Control Difficulties. The groups did not differ significantly on any other subscales.

The finding that adolescents with BPD had higher overall self-reported emotion dysregulation compared with psychiatric controls and healthy controls is consistent with theoretical conceptualizations that suggest that BPD is a central feature of the disorder [27, 28] as well as previous studies with adults and adolescents which have shown that those with BPD symptomatology demonstrate greater emotion dysregulation than healthy controls [31, 47]. It is important to note that some more recent studies contradict these findings, suggesting that difficulties in emotion regulation are not specific to BPD [48–50], though these studies were conducted with adults. Though a previous study did examine the relationship between emotion dysregulation and psychiatric severity in adolescents [41], it did not examine patients with BPD and compare them to other groups. Our findings showed that adolescents with BPD had higher overall self-reported emotion dysregulation than both psychiatric controls and healthy controls, even after controlling for psychiatric severity. This suggests that differences among groups were not simply an artifact of higher psychiatric severity, or distress, among the BPD group. Instead, it suggests that problems with emotion regulation may be a BPD-specific feature, central to the pathology of the disorder.

Within specific DERS subscales, our second finding demonstrated that adolescents with BPD had higher self-reported scores than both groups on limited access to strategies and impulse control difficulties. These subscales may be conceptualized as capturing the behavioral subscales of the DERS, reflecting the components of emotion regulation that relate to capacity to flexibly manage emotions using a variety of strategies (i.e. limited access to strategies) and regulate one’s behavior when experiencing intense emotions (i.e. impulse control difficulties). Indeed, elevations on both subscales capture aspects of the disorder that have been well documented in theoretical and empirical literature. Namely, when individuals with BPD are emotionally distressed, they struggle to flexibly use emotion regulation strategies [51] and manage impulsive behavior. In addition, previous studies acknowledge that impulsivity is a core feature of BPD [52, 53], as those with BPD tend to favor immediate gratification over long-term reward [54]. Indeed, research has demonstrated that aggression [55], drug dependence [56], self-injury [57], and other behavioral problems [58], are common in the disorder. These findings also empirically support how evidence-based interventions for BPD treat the disorder. For example, dialectical behavior therapy (DBT, [28]) assumes that individuals with BPD have emotion regulation skills (or strategies) deficits and therefore targets problem behaviors by providing patients with myriad coping strategies so that they may flexibly access these strategies in their daily lives. As a result, DBT has been successful in reducing impulsive behavior in individuals with the disorder [59, 60]. Thus, our findings that those with BPD have higher self-reported scores than both groups on the DERS subscales of limited access to strategies and impulse control difficulties are consistent with our current understanding of the phenomenology and treatment of BPD and with a current method of BPD treatment.

Beyond the aforementioned subscales, groups did not differ significantly on subscales of lack of awareness of emotional responses, lack of clarity of emotional responses, non-acceptance of emotional responses, and difficulties engaging in goal-directed behaviors when experiencing negative emotions. This may reflect the fact these subscales may capture emotion regulation difficulties found in other disorders, not necessarily those specific to BPD. For example, individuals with generalized anxiety disorder demonstrate difficulty with emotional awareness [61], clarity [62, 63], non-acceptance [64], and difficulty engaging in goal-directing behavior [63]. In addition, theoretical [27, 28] and empirical [52] work identifies difficulties in managing behavior as a core feature of BPD—one that distinguishes it from internalizing disorders (e.g., depression, anxiety) in which difficulties with emotions is also core to the psychopathology. It is also possible that subscales that did not produce significant differences were worded in such a way that failed to capture the unique emotion regulation difficulties that individuals with BPD experience. For example, an item from the Lack of Emotional Awareness scale states, “I pay attention to how I feel (reverse scored).” Though this item is meant to capture an aspect of mindfulness inherent to emotion regulation, is possible that this item was misinterpreted by individuals with BPD as capturing the more ruminative aspects of emotional distress. Thus, the use of additional measures of emotion dysregulation to disentangle these relations would be useful in this regard.

On the other hand, though groups did not differ significantly on subscales of lack of awareness of emotional responses, lack of clarity of emotional responses, non-acceptance of emotional responses, and difficulties engaging in goal-directed behaviors when experiencing negative emotions, absence of evidence does not necessarily imply evidence of absence. Upon examination of effect sizes for these specific contrasts, the BPD participants and healthy controls scales of non-acceptance of emotional response and difficulties engaging in goal directed behaviors were of medium magnitude. Again, future research could further elucidate these findings by including additional measures of emotion dysregulation, as mentioned previously.

The impact of the findings of the present study is limited by several factors. Notably, the use of a self-report measure, like the DERS, renders participant responses susceptible to the effects of retrospective recall bias [65]. Moreover, given that emotions in individuals with BPD are highly variable, unstable, and reactive [28, 66], use of a one-occasion assessment of emotion dysregulation may fail to fully and accurately capture emotion dysregulation in this particular population. Recognizing the limits of traditional self-report assessment of BPD mood, there has been a recent push to use intensive longitudinal designs (e.g., ecological momentary assessment (EMA); [67]) to study affect problems in BPD, though it is worth noting that nearly all studies focused on adult samples (see [68] for a review), save for a few (i.e., [69, 70]). Future research should not only investigate this with ecological momentary assessment, but it should also make use of behavioral measures of emotion regulation in adolescents to complement these findings. Second, the clinical samples from this study consisted of mostly Caucasian adolescents of high socioeconomic status, and thus the generalizability of our findings is limited. Moreover, the healthy and clinical groups were not demographically matched; the healthy control group was comprised of racially, ethically, and socioeconomically diverse adolescents. Future studies should match groups on socioeconomic status, race, and ethnicity or statistically control for these variables in analyses. Third, it is possible that findings of higher levels of emotional dysregulation in BPD participants were due to differential levels of trust or confidence in self-reported emotion regulation capacities, such that individuals with BPD may have experienced less trust overall in their emotion regulation abilities compared with the other groups. Thirdly, within the DERS, 11 out of the 34 items are recoded in a positive direction. Given that there are more negatively phrased items than positively phrased ones, it is possible that findings were influenced by answering biases. Including additional measures of emotion dysregulation, as previously mentioned, would likely attenuate the potential effects of such a bias. In addition, our study followed one particular model of emotion dysregulation (i.e. [9]). However, there are undoubtedly many other models of emotion dysregulation (e.g., experiential avoidance model, [71, 72]), and therefore, future studies may want to consider other conceptualizations of emotion dysregulation when examining emotion dysregulation in adolescents with BPD. Our study utilized only one measure of emotion dysregulation (i.e. the DERS). In order to further support findings of group differences in emotion regulation difficulties, future studies should make use of multiple measures of this construct. Finally, though the current study demonstrated differences in self-reported emotion regulation among BPD vs. psychiatric controls, we did not investigate differences between adolescents with BPD and specific diagnoses within the psychiatric control group. Indeed, though emotion dysregulation has been shown to be a construct that cuts across multiple forms of psychopathology, the degree to which it differentially relates to BPD versus specific disorders is less clear, especially in adolescents. While future research should make use of designs that contrast BPD with unique disorders in adolescents to elucidate these relations, we also acknowledge that high comorbidity is the rule rather than the exception among adolescents with severe psychopathology, suggesting that lumping (instead of splitting) disorders may be clinically more meaningful.

## Conclusions

Notwithstanding these limitations, this study was the first to compare adolescents with BPD to psychiatric controls and healthy controls on emotion dysregulation, demonstrating that emotion dysregulation in general, as well as limited access to strategies and problems with impulsivity, more specifically, represent particularly elevated emotion dysregulation difficulties specific to adolescents with BPD. These findings support current conceptualizations of BPD as representing problems with behavioral management and coping skills when emotionally distressed. It also provides empirical support for current interventions for the disorder, validating the need to target impulse control difficulties and enhance regulation strategies in those with the disorder.
